# Prognostic models based on postoperative circulating tumor cells can predict poor tumor recurrence-free survival in patients with stage II-III colorectal cancer

**DOI:** 10.7150/jca.30512

**Published:** 2019-07-25

**Authors:** Dong Wang, Yingchi Yang, Lan Jin, Jin Wang, Xiaomu Zhao, Guocong Wu, Jinghui Zhang, Tiankuo Kou, Hongwei Yao, Zhongtao Zhang

**Affiliations:** Department of General Surgery, Beijing Friendship Hospital, Capital Medical University, Beijing Key Laboratory of Cancer Invasion and Metastasis Research & National Clinical Research Center of Digestive Diseases, No. 95 Yongan Road, Xi-Cheng District, Beijing, 100050, China.

**Keywords:** circulating tumor cells, non-metastatic colorectal cancer, tumor recurrence survival, prognostic model

## Abstract

**Background:** It is urgent to develop robust prognostic biomarkers for non-metastatic colorectal cancer (CRC) patients undergoing surgery. The current study aimed to explore and compare the clinical significance of preoperative and postoperative blood tumor biomarkers including circulating tumor cells (CTCs), and develop prognostic models based on tumor biomarkers in patients with stage II-III CRC receiving surgery.

**Methods:** A prospective study was performed to enroll 130 patients with stage II-III CRC receiving surgery between January 2015 and December 2017. Preoperative and postoperative blood tumor biomarkers including CTCs were detected and their prognostic value in predicting tumor recurrence-free survival (RFS) in stage II-III CRC were identified by Kaplan-Meier curves and Cox proportional hazard regression models.

**Results:** CTCs counts within three postoperative days were significantly higher than preoperative CTCs (pre-CTCs). No significant association of pre-CTCs with clinical characteristics and tumor biomarkers was observed while positive postoperative CTCs (post-CTCs) were associated with female, older onset age, high TNM stage, tumor recurrence, and preoperative CEA. Kaplan-Meier curve with log-rank test and univariate Cox proportional hazard regression analysis suggested high N stage, TNM stage, positive pre-carbohydrate antigen (CA) 125, pre-CA19-9, post-CA125, post-CA19-9, post-CA72-4, post-carcinoembryonic antigen (CEA), and post-CTCs were correlated with poor RFS. In multivariate analysis, only TNM stage (adjusted HR=3.786, 95% CI=1.330-10.780; P=0.013), post-CA72-4 (adjusted HR=5.675, 95% CI=2.064-15.604; P=0.001), and post-CTCs (adjusted HR=2.739, 95% CI=1.042-7.200; P=0.041) were significantly correlated with poor RFS. We then developed prognostic models combining post-CTCs and post-CA72-4 with TNM stage or not to stratify the patients into different risk groups. These prognostic models exert a similar good performance in predicting tumor RFS in stage II-III CRC patients.

**Conclusions:** Postoperative CTCs were prior to preoperative CTCs in predicting tumor recurrence survival in non-metastatic CRC patients undergoing surgery. We also developed CTCs-based prognostic models to predict tumor recurrence in stage II-III CRC, which might be used to identify the patients with high risk of recurrence and guide aggressive treatment to improve the clinical outcomes of those patients.

## Introduction

Colorectal cancers (CRC) are the fourth most commonly diagnosed cancer and the second leading cause of cancer-related deaths around the world according to the GLOBOCAN 2018 estimate^1^. Surgery with or without other adjuvant therapy is the common method in treatment of CRC patients especially for those with non-metastatic colorectal cancer (non-mCRC, UICC stage I-III)^2^. Along with the progress in diagnosis and therapy, patients with CRC obtain a decreasing mortality, however, the 5-year overall survival (OS) rate of non-mCRC patients is still low and approximately 25-50% of patients with stage II-III CRC develop recurrence and metastasis after comprehensive treatment^3^.

The mechanisms of recurrence and metastasis of CRC may involve a series of cell biological behaviors, including circulating tumor cells (CTCs), which are cells migrating from solid tumors into the peripheral blood and play an important role in the process of distant metastasis based on the “seed and soil theory” 4. Recently, CTCs has been widely proposed to serve as biomarkers in various cancer types including breast^5^, ^6^, prostate^7^, and colorectal^8^, ^9^ cancers. In non-mCRC patients, preoperative detected CTCs is a valid prognostic factor for cancer progression and survival^10^. In patients with primary CRC undergoing surgical resection, CTCs is associated with an increased risk of postoperative metastasis^11^. Their persistent presence after curative resection is also found to be associated with poor prognosis^12^, however, related studies comparing clinical significance of preoperative and postoperative CTCs in non-mCRC are rare. Thus, it is an urgency to compare the prognostic clinical significance of preoperative and postoperative CTCs in non-mCRC and investigate the potential prognostic models based on pre- or postoperative CTCs.

Currently, we performed a prospective observational study in a single institute to recruit patients with stage II-III CRCs, explore and compare the clinical significance of preoperative and postoperative blood tumor biomarkers including CTCs, and develop prognostic models based on tumor biomarkers.

## Materials and methods

### Patients

This was a prospective observational study at a single institute to recruit operable stage II-III CRC patients according to the seventh edition of TNM/The Union for International Cancer Control (UICC)/American Joint Committee on Cancer (AJCC) classification from Beijing Friendship Hospital between January 2015 and December 2017. Inclusion criteria: (1) patients with primary stage II-III colorectal cancers; (2) the tumors of the patients were operable and then resected in our hospital. Exclusion criteria: (1) patients had received radiotherapy and/or chemotherapy prior to surgery; (2) patients with hematologic, hepatic, autoimmune diseases, recent infection, or other malignancies. According to the inclusion and exclusion criteria, a total of 130 patients were included from our hospital. Basel clinical characteristics of the patients were collected and follow-up of tumor recurrence was performed every three months up to May 2018. Blood samples before operation and within three days after operation were collected. Written informed consent was obtained from all patients and all procedures were approved by the Institutional Research Ethics Committee.

### Measurement of peripheral circulating tumour cells

CTCs detection was performed via a protocol described in our previous study^13^. Briefly, cells in 3.2 ml blood sample were collected by centrifugation at 650 × *g* at room temperature and resuspended in a lysis buffer solution to remove red blood cells, and contrifugated at 650 × *g* at room temperature. The residual cell pellet was resuspended in phosphate buffer solution and subsequently incubated with anti-CD45 monoclonal antibody-coated magnetic beads for 30 min, followed by the separation of magnetic beads using a magnetic stand (Promega, Madison, WI, USA). Afterwards, the samples were centrifuged at 800 × *g* for 3 min and spotted on glass slides to analyze enriched CTCs by immuno-fluorescence *in situ* hybridization (*imFISH*) staining (27). In addition, traditional tumor markers in plasma samples including CEA, CA19-9, CA125, and CA72-4 were detected by an electrochemiluminescence immunoassay (Cobas E601; Roche, Indianapolis, IN, U.S.A.) according to the manufacturer's instructions.

### Statistical analysis

The patients' demographic data was summarized as the number (%) for categorical variables, and the median, 95% confidence interval (CI), and range for continuous variables. Levels of preoperative and postoperative tumor markers including CTCs, CEA, CA19-9, CA72-4, and CA125 were compared by paired student's T test. Correlations among the preoperative and postoperative CEA, CA19-9, CA72-4, and CA125 were evaluated by Pearson's correlation coefficients. The optimal cutoff points of preoperative and postoperative CEA, CA19-9, CA72-4, and CA125 were determined by X-tile software version 3.6.1 (Yale University, New Haven, CT, USA) based on tumor recurrence-free survival (RFS). Categorical variables in different groups were compared by Chi-square test or Fisher's exact test. Prognostic effects of clinical parameters and tumors markers on RFS were assessed by Kaplan-Meier Curves and Univariate and multivariate Cox proportional hazard regression. Variables with a P value < 0.05 in the univariate analysis were included for analysis in the multivariate model. Independent prognostic factors obtained from Multivariate Cox proportional hazard model analysis were used to construct prognostic scores. Harrell's concordance index (c-index) were calculated by R 3.3.2 software (Institute for Statistics and Mathematics, Vienna, Austria) to determine the predictive accuracy of the prognostic models. Patients were further stratified into two prognostic groups according to their total score obtained from the prognostic model. Fisher's exact test, Chi-square test, and Pearson's correlation analyses were conducted via GraphPad Prism 6.0 software (La Jolla, CA, USA). Survival curves were generated by MedCalc version 15.2 software. Univariate and multivariate logistic regression were performed using SPSS 19.0 software. All tests were two-sided. A P value of less than 0.05 was considered significant.

## Results

### Baseline characteristics of patients

A total of 130 stage II-III CRC patients were recruited in the prospective observational study (Table S1). The median age of the patients was 63.00 years. 74 cases were male and 54 were female. 47 cases located in rectum and 83 cases in colon. The cases with T1-2 and T3-4 depth were 20 and 110, respectively. Lymph-node metastasis was observed in 53 patients. 75 cases were with stage II disease and 55 were with stage III disease. 12 cases were poor differentiated while 116 were moderate or well differentiated. During the follow-up period, tumor recurrence occurred in 18 patients, 112 patients with no recurrence, and ten patients were lost.

### Comparison of pre- and postoperative CTCs, CA125, CA19-9, CA72-4 and CEA

**Figure 1:**
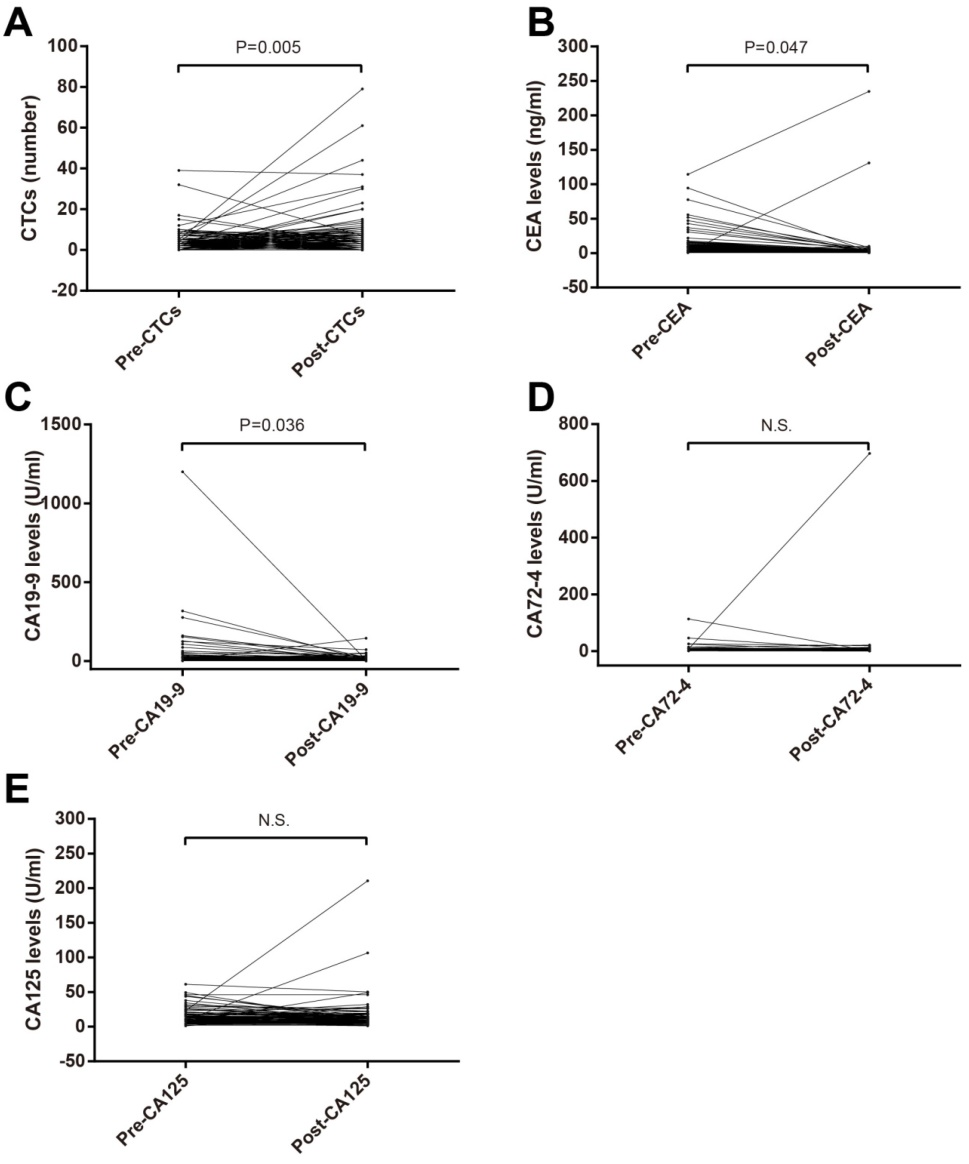
Comparison of preoperative and postoperative blood tumor biomarkers in patients with stage II-III CRC. (A) CTCs; (B) CEA; (C) CA19-9; (D) CA72-4; (E) CA125. Abbreviations: CRC, colorectal cancer; CTCs, circulating tumor cells; CEA, carcinoembryonic antigen; CA19-9, carbohydrate antigen 19-9; CA72-4, carbohydrate antigen 72-4; CA125, carbohydrate antigen 125.

Paired T test were used to compare pre- and postoperative CTCs, CA125, CA19-9, CA72-4 and CEA. CTCs number in postoperation was bigger than that in preoperation (Figure 1A) while postoperative CEA (Figure 1B) and CA19-9 (Figure 1C) levels were lower. No significant difference of CA72-4 and CA125 between in preoperation and postoperation was observed (Figure 1D and E).

### Correlation of preoperative and postoperative CTCs levels with clinical characteristics

**Figure 2:**
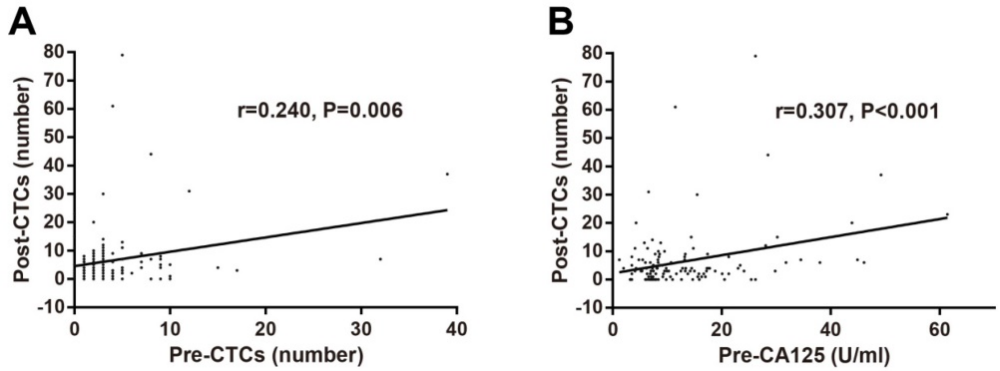
Correlations of postoperative CTCs numbers with preoperative CTCs numbers (A) and preoperative CA125 levels (B) in stage II-III CRC determined by Pearson's correlation analysis. Abbreviations: CRC, colorectal cancer; CTCs, circulating tumor cells; CA125, carbohydrate antigen 125.

The optimal cut-off values of preoperative and postoperative CTCs levels and other tumor biomarkers were identified using X-tile software based on RFS. The correlations of preoperative and postoperative CTCs with clinical characteristics including gender, age, tumor location, T stage, N stage, TNM stage, tumor differentiation, tumor recurrence, and traditional tumor biomarkers were determined by Chi-square test. No significant association of preoperative CTCs with clinical characteristics and tumor biomarkers was observed (Table 1). As for postoperative CTCs, positive CTCs were associated with female, older onset age, high TNM stage, tumor recurrence, and preoperative CEA (Table 2). In addition, correlations among pre/postoperative tumors biomarkers were investigated and postoperative CTCs levels was found to be associated with preoperative CTCs levels (r=0.240, P=0.006; Figure 2A) and preoperative CA125 levels (r=0.307, P<0.001; Figure 2B).

### Prognostic roles of clinical characteristics and pre/postoperative tumor biomarkers in stage II-III CRC

**Fig 3:**
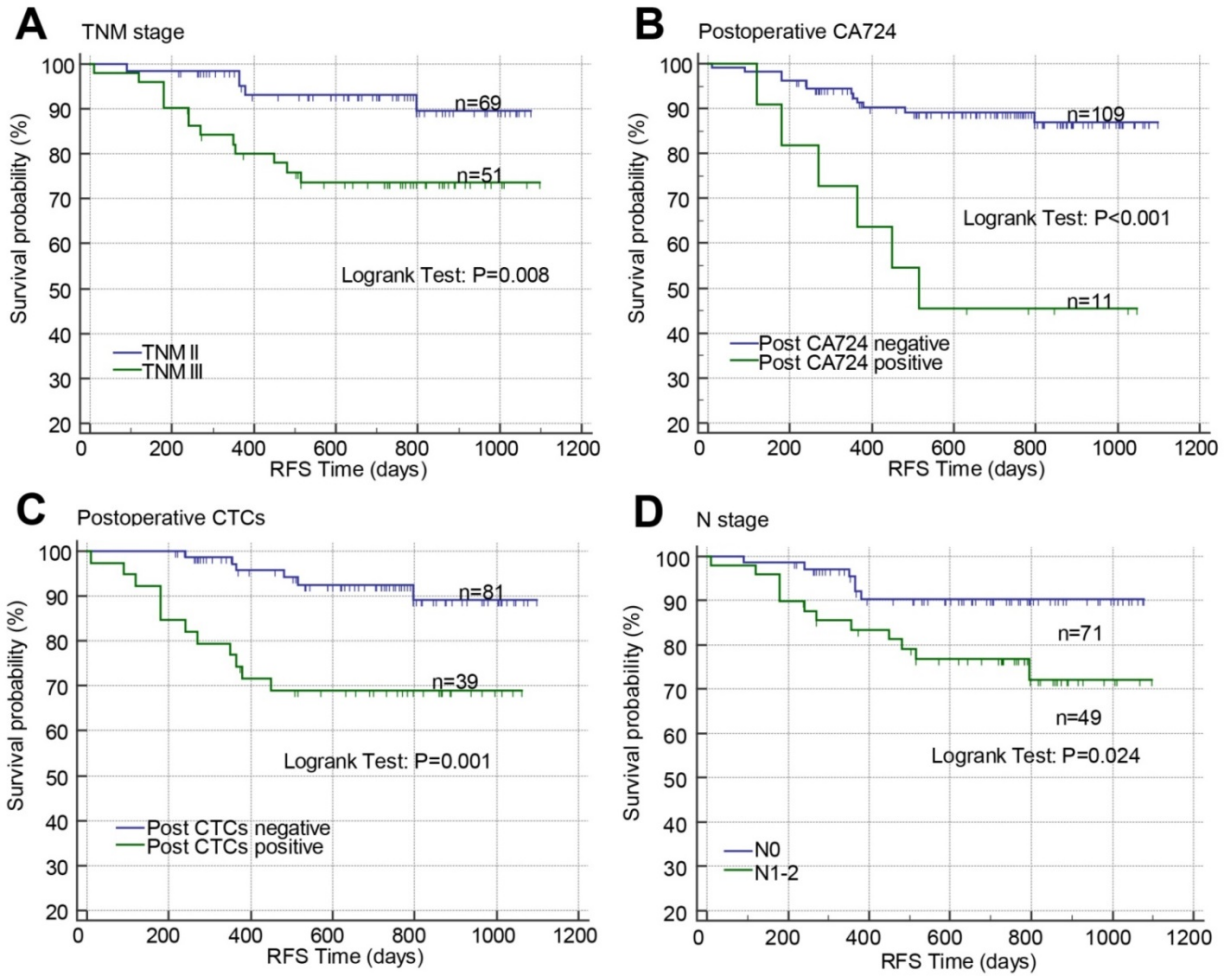
Effects of TNM stage (A), postoperative CA72-4 (B), postoperative CTCs (C), and N stage (D) on RFS in stage II-III CRC analyzed by Kaplan-Meier curves. Abbreviations: CRC, colorectal cancer; CTCs, circulating tumor cells; RFS, recurrence-free survival; CA72-4, carbohydrate antigen 72-4; RFS, recurrence-free survival.

Kaplan-Meier curves with log-rank test and Cox proportional hazard regression analysis were used to investigate the prognostic effects of the baseline clinical characteristics and pre-/post-operative CTCs, CA125, CA19-9, CA72-4, and CEA on RFS in patients with stage II-III CRC. In Kaplan-Meier curves, high TNM stage (P=0.008; Figure 3A), positive postoperative CA72-4 (P<0.001; Figure 3B), postoperative CTCs (P=0.001; Figure 3C), and N stage (P=0.024; Figure 3D) were associated with poor RFS. In univariate Cox proportional hazard regression analysis, high N stage (crude HR=2.879, 95% CI=1.080-7.672; P=0.034), TNM stage (crude HR=3.674, 95% CI=1.309-10.306; P=0.013), positive pre-CA125 (crude HR=2.886, 95% CI=1.138-7.318; P=0.026), pre-CA19-9 (crude HR=3.226, 95% CI=1.149-9.056; P=0.026), post-CA125 (crude HR=2.793, 95% CI=1.074-7.264; P=0.035), post-CA19-9 (crude HR=3.165, 95% CI=1.038-9.649; P=0.043), post-CA72-4 (crude HR=5.589, 95% CI=2.096-14.904; P=0.001), post-CEA (crude HR=3.821, 95% CI=1.247-11.705; P=0.019), and post-CTCs (crude HR=4.435, 95% CI=1.664-11.820; P=0.003) were correlated with poor RFS (Table 3). In multivariate analysis, only TNM stage (adjusted HR=3.786, 95% CI=1.330-10.780; P=0.013), post-CA72-4 (adjusted HR=5.675, 95% CI=2.064-15.604; P=0.001), and post-CTCs (adjusted HR=2.739, 95% CI=1.042-7.200; P=0.041) were significantly correlated with poor RFS (Table 3). Due to post-CA72-4 was associated with RFS, the correlations of pre/post-CA72-4 with clinical characteristics and tumor biomarkers were determined by Chi-square test or Fisher's exact test. Pre-CA72-4 was found to be correlated with post-CA72-4 while post-CA72-4 was correlated with post-CEA, post-CA125, and tumor recurrence (Table S2 and S3).

### Construction of the prognostic models based on postoperative CTCs in stage II-III CRC

**Figure 4:**
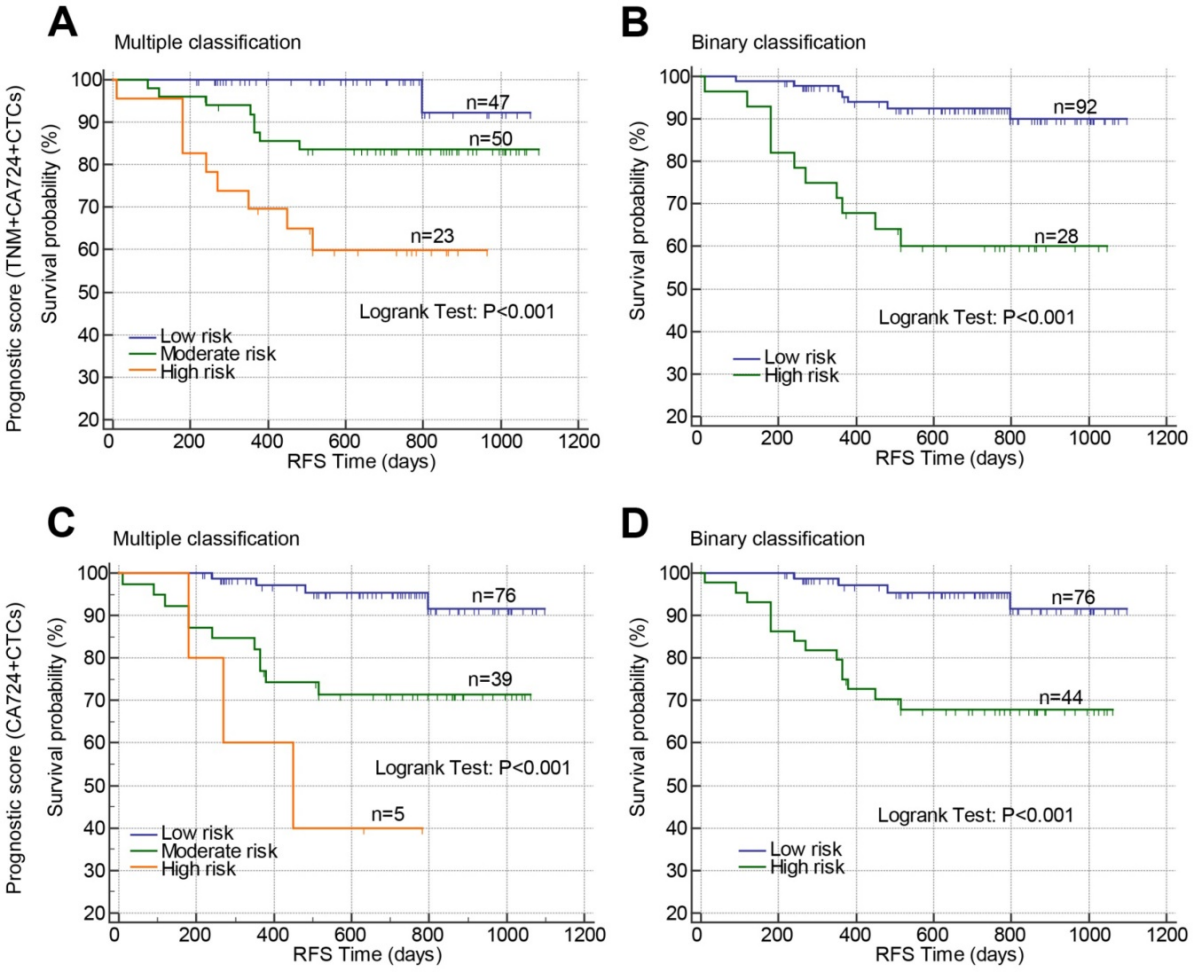
Prognostic value of constructed prognostic models based on postoperative CTCs in stage II-III CRC. Prognostic models were constructed by combining TNM stage, postoperative CA72-4 and postoperative CTCs (A and B) or combining only postoperative CA72-4 and postoperative CTCs (C and D). Using the prognostic models, the patients were stratified into three risk groups (multiple classifications, A and C) or two risk groups (binary classification, B and D). Abbreviations: CRC, colorectal cancer; CTCs, circulating tumor cells; CA72-4, carbohydrate antigen 72-4; RFS, recurrence-free survival.

Above results suggested that TNM stage, post-CA72-4, and post-CTCs were independent prognostic factors for RFS in stage II-III CRC, which were used to develop prognostic models. The β-coefficients from the multivariate Cox proportional hazard regression analysis were used to generate the risk points of the prognostic factors. Scores 3, 4, and 5 were assigned to positive post-CTCs, stage III, and positive post-CA72-4 in the scoring system based on the β-coefficients. The total prognostic scores of the risk model containing TNM stage, post-CTCs, and post-CA72-4 ranged from 0 to 12 (Prognostic model 1). Using the prognostic scores, the patients were stratified into three risk groups (mutiple classification: low risk: score 0; moderate risk: score 3-5; high risk: score 7-12). Comparing the low risk group, the hazard ratios of moderate and high risk groups were 6.810 and 20.053, respectively (Figure 4A and Table 4). The c-index was 0.760 (95%CI: 0.633-0.887). Then the patients were stratified into two risk groups (Binary classification: Low risk: score 0-4; High risk: score 5-12). The hazard ratio was 5.894 when comparing the high and low risk groups with a c-index of 0.720 (95%CI: 0.622-0.818) (Figure 4B and Table 4). In addition, we constructed a prognostic model that only comprised post-CTCs and post-CA72-4 with a total score ranging from 0 to 8 (Prognostic model 2). Using the prognostic model 2, the patients were divided into multiple classification (Low, moderate and high risk groups) and binary classification (Low and high risk groups). In multiple classification, comparing the low risk group, the hazard ratios of moderate and high risk groups were 5.429 (adjusted HR=5.289) and 14.427 (adjusted HR=14.111), respectively, with the c-index of 0.748 (Figure 4C and Table 4). In binary classification, the hazard ratio was 6.279 (adjusted HR=6.092) when comparing the high and low risk groups with a c-index of 0.732 (Figure 4D and Table 4). To further investigated the predictive value of the prognostic models in patient tumor recurrence, their accuracy in predicting tumor recurrence was also analyzed.

In prognostic model 1, the tumor recurrence rate in low risk and high risk groups was 7.61% and 39.29%, respectively, with an accuracy rate of 80%, AUC of 0.722, sensitivity of 61.11%, and specificity of 73.53% (Table 5 and 6). In prognostic model 2, the tumor recurrence rate in low risk and high risk groups was 5.56% and 31.82%%, respectively, with an accuracy rate of 71.67%, AUC of 0.742, sensitivity of 77.78%, and specificity of 70.59% (Table 5 and 6). Therefore, our finding suggested that the two prognostic models showed a similar performance in predict RFS and tumor recurrence in stage II-III CRC patients.

### Validation of the prognostic models based on postoperative CTCs in stage II-III rectal cancer or colon cancer

**Figure 5:**
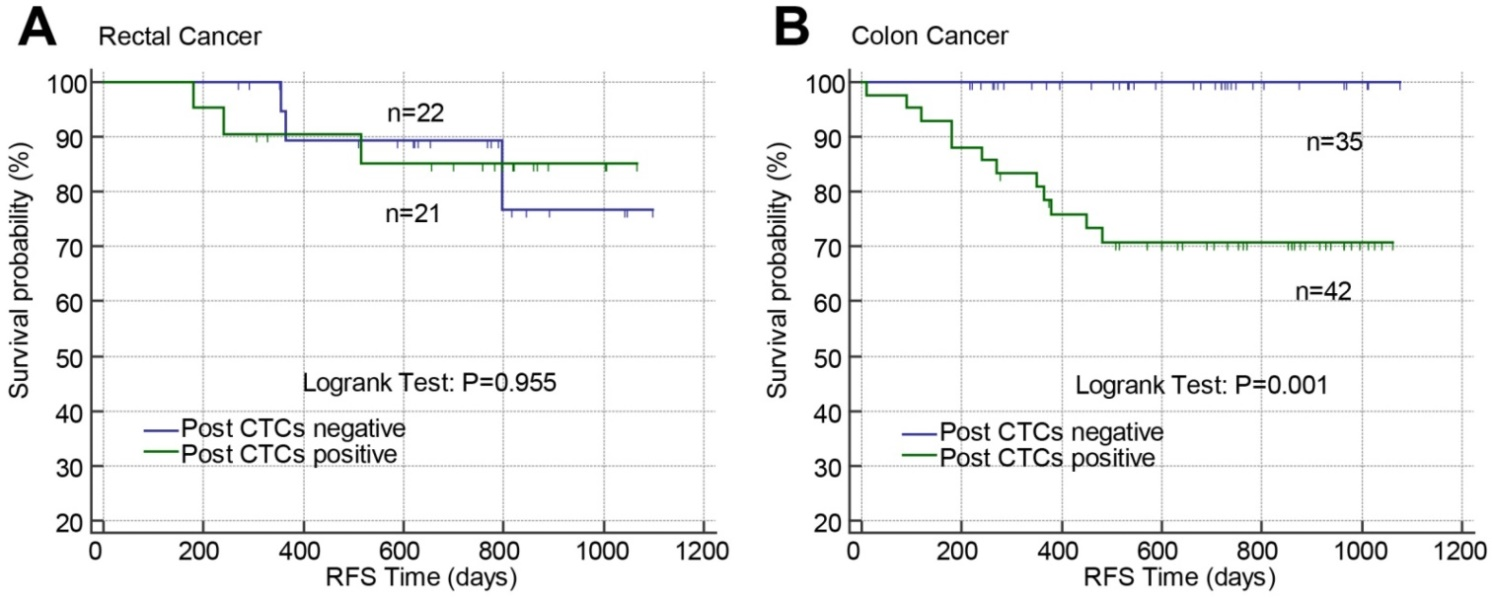
Effects of postoperative CTCs on RFS in stage II-III rectal cancer (A) or colon cancer (B) analyzed by Kaplan-Meier curves. Abbreviations: CTCs, circulating tumor cells; RFS, recurrence-free survival.

**Figure 6:**
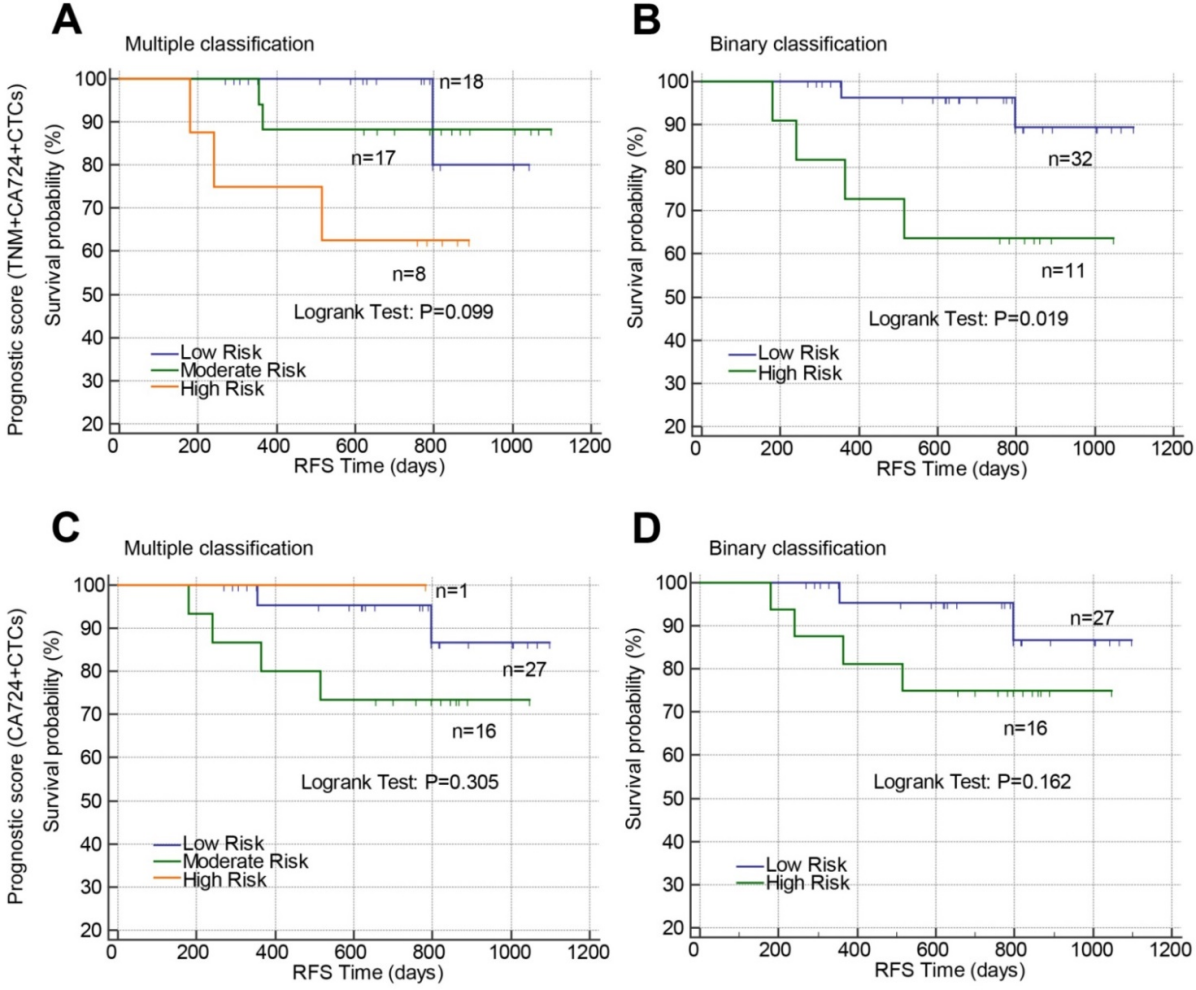
Prognostic value of constructed prognostic models based on postoperative CTCs in stage II-III rectal cancer. Prognostic models were constructed by combining TNM stage, postoperative CA72-4 and postoperative CTCs (A and B) or combining only postoperative CA72-4 and postoperative CTCs (C and D). Using the prognostic models, the patients were stratified into three risk groups (multiple classifications, A and C) or two risk groups (binary classification, B and D). Abbreviations: CTCs, circulating tumor cells; CA72-4, carbohydrate antigen 72-4; RFS, recurrence-free survival.

The predictive value of post-CTCs and the constructed prognostic models were validated according to tumor location (rectum or colon). Post-CTCs were only associated with RFS in colon cancer but not rectal cancer (Figure 5). In rectal cancer, only when the patients were classified into high risk and low risk by prognostic model 1 (combining TNM stage, post-CTCs, and Post-CA-72-4; Binary classification), significant difference of RFS between high risk and low risk patients was found (Figure 6B; P=0.019).

**Fig 7:**
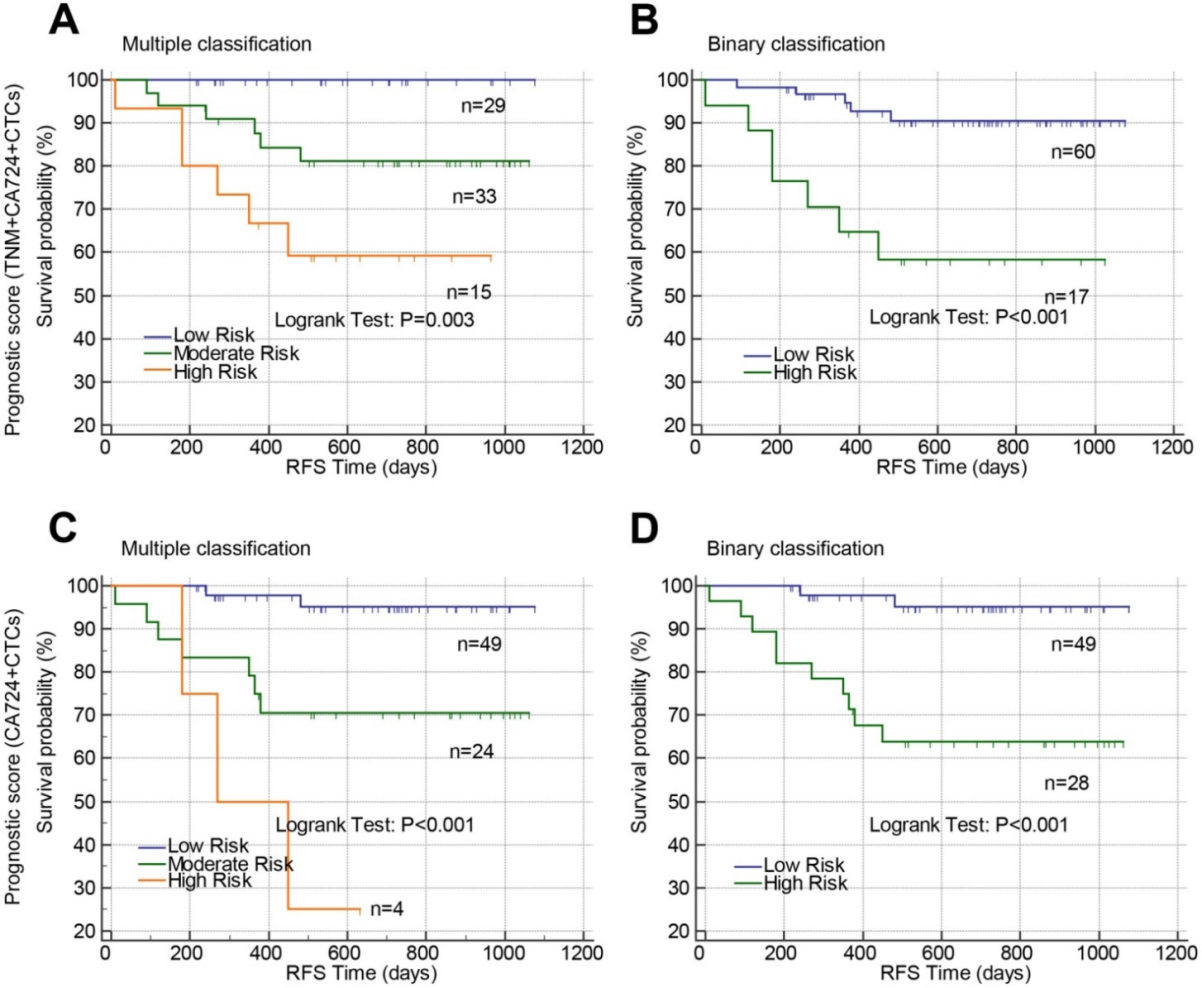
Prognostic value of constructed prognostic models based on postoperative CTCs in stage II-III colon cancer. Prognostic models were constructed by combining TNM stage, postoperative CA72-4 and postoperative CTCs (A and B) or combining only postoperative CA72-4 and postoperative CTCs (C and D). Using the prognostic models, the patients were stratified into three risk groups (multiple classifications, A and C) or two risk groups (binary classification, B and D). Abbreviations: CTCs, circulating tumor cells; CA72-4, carbohydrate antigen 72-4; RFS, recurrence-free survival.

In colon cancer, the patients were well classified by prognostic model 1 (Figure 7A, P=0.003 and Figure 7B, P<0.001) and prognostic model 2 (Figure 7C, P<0.001 and Figure 7D, P<0.001) into either three risk groups (Figure 7A and C) or two groups (Figure 7B and D). The accuracy of the prognostic models in predicting tumor recurrence in rectal cancer or colon cancer was also analyzed by receiver operating curves (Table 7). Prognostic model 1 exhibited high accuracy in rectal cancer with an AUC of 0.732 while Post-CTCs, prognostic model 1 and 2 all exhibited high accuracy in colon cancer with AUC of 0.769, 0.715, and 0.778, respectively.

## Discussion

A series of studied have evaluated the prognostic significance of postoperative CTCs in non-metastatic colorectal cancer^14^-^21^. A recent meta-analysis performed by Lu et al in 2017 revealed the associations of prognosis with both preoperative and postoperative CTCs in non-mCRC^9^. However, the studies that compared preoperative and postoperative CTCs in the same CRC patients were rare and the results were conflicting. Krust et al found postoperative but not preoperative CTCs was associated with both progression-free survival and overall survival in CRC^17^. Galizia et al detected CTCs before and at 1 month after surgery and only found the association of postoperative level of CTCs with tumor recurrence in CRC patients undergoing surgical resection^22^. Van Dalum et al determined CTCs prior to surgery, at weeks and 2-3 years after surgery in non-mCRC and found CTCs prior to surgery and on 2-3 years after surgery but not CTCs at weeks after surgery were associated with tumor recurrence^21^. In our study, we prospectively enrolled 130 patients with stage II-III CRC patients and found positive postoperative CTCs were correlated with poor RFS (crude HR=4.435, 95% CI=1.664-11.820; P=0.003; adjusted HR=2.739, 95% CI=1.042-7.200; P=0.041) while found no difference of RFS in patients with positive or negative preoperative CTCs. Lu et al suggested that persistent postoperative CTCs (lasting for 4 weeks postoperatively) was associated with early relapse in stage II-III colon cancer patients^18^. Based on the previous and our current studies, we speculated that the timing of CTCs detection is crucial and positive CTCs before, during or after surgery may have different biological and clinical significance. And postoperative CTCs might better reflect the most relevant CTCs status because it combines preoperative status, intraoperative blood spreading, and rapid apoptotic death of shed cells^23^.

Up to date, rare study has been carried out to develop potential prognostic models based on blood tumor biomarkers and other clinicopathological features to predict clinical outcomes of CRC patients. Chou et al have enrolled prospectively 55 mCRC patients and established a reliable CTCs-based prognostic model combining CTCs at baseline of palliative chemotherapy, TNM stage, ECOG performance, histological grade and previous history of colectomy for the prediction of clinical outcomes in mCRC patients treated with chemotherapy 24. For non-mCRC, we, for the first time, developed prognostic models combining postoperative CTCs, CA72-4 with or without TNM stage, which might be useful supplementary tools in detecting early relapse and survival rate of stages II-III CRC patients undergoing curative surgery.

There are several inherent limitations in the present study. Firstly, the sample size was limited and the study was only performed in a single medical institute. Secondly, the therapeutic strategy information after surgery including adjuvant chemotherapy in the study was not complete and not included in statistical analyses. Finally, although the accuracy of our constructed prognostic models was evaluated by c-index, external validations were lacked. Thus, there is a need for large, well-designed prospective trials to verify the clinical significance of our prognostic models in postoperative early relapse in CRC patients.

In conclusion, we performed a prospective study to compare prognostic value of preoperative and postoperative CTCs in CRC recurrence and identified postoperative but not preoperative CTCs was independent factor in predicting tumor recurrence in stage II-III CRC. Furthermore, we developed reliable prognostic models based on postoperative CTCs, postoperative CA72-4 with or without TNM stage for the predication of tumor recurrence in non-mCRC. These models might be used to identify the patients with high risk of recurrence and guide aggressive treatment to improve the clinical outcomes of those patients.

## Supplementary Material

Supplementary tables.

## Figures and Tables

**Table 1:** Associations of preoperative CTCs with basal characteristics, pre- and postoperative tumor biomarkers, and tumor recurrence in stage II-III CRC

Parameters	Subgroups	Preoperative CTCs		χ2	P-value
		Negative	Positive		
**Gender**	Male	41	35	0.739	0.390
	Female	25	29		
**Age**	≤62 yrs	34	27	1.135	0.287
	>62 yrs	32	37		
**Tumor location**	Rectum	27	20	1.313	0.252
	Colon	39	44		
**T stage**	T1-2	8	12	1.097	0.295
	T3-4	58	52		
**N stage**	N0	37	40	0.558	0.455
	N1-2	29	24		
**TNM stage**	II	36	39	0.544	0.461
	III	30	25		
**Tumor differentiation**	Poor	5	7	0.519	0.471
	Moderate/well	61	55		
**Preoperative CA125**	Negative	53	47	0.601	0.438
	Positive	13	16		
**Preoperative CA19-9**	Negative	58	57	0.045	0.833
	Positive	8	7		
**Preoperative CA72-4**	Negative	58	57	1.147	0.284
	Positive	8	4		
**Preoperative CEA**	Negative	44	46	0.414	0.520
	Positive	22	18		
**Postoperative CA125**	Negative	36	36	0.412	0.521
	Positive	16	12		
**Postoperative CA19-9**	Negative	62	55	2.312	0.128
	Positive	4	9		
**Postoperative CA72-4**	Negative	58	61	2.318	0.128
	Positive	8	3		
**Postoperative CEA**	Negative	59	59	0.303	0.582
	Positive	7	5		
**Tumor recurrence**	No recurrence	52	50	0.630	0.427
	Recurrence	11	7		

Abbreviations: CRC, colorectal cancer; CTCs, circulating tumor cells; CEA, carcinoembryonic antigen; CA125, carbohydrate antigen 125; CA19-9, carbohydrate antigen 19-9; CA72-4, carbohydrate antigen 72-4.

**Table 2:** Associations of postoperative CTCs with basal characteristics, pre- and postoperative tumor biomarkers, and tumor recurrence in stage II-III CRC

Parameters	Subgroups	Postoperative CTCs		χ2	P-value
		Negative	Positive		
**Gender**	Male	44	32	6.519	**0.011**
	Female	19	35		
**Age**	≤62 yrs	40	21	13.474	**<0.001**
	>62 yrs	23	46		
**Tumor location**	Rectum	25	22	0.659	0.417
	Colon	38	45		
**T stage**	T1-2	10	10	0.022	0.881
	T3-4	53	57		
**N stage**	N0	42	35	2.799	*0.094*
	N1-2	21	32		
**TNM stage**	II	43	32	5.586	**0.018**
	III	20	35		
**Tumor differentiation**	Poor	5	7	0.243	0.622
	Moderate/well	57	59		
**Preoperative CA125**	Negative	52	48	2.763	*0.096*
	Positive	10	19		
**Preoperative CA19-9**	Negative	59	56	3.225	*0.073*
	Positive	4	11		
**Preoperative CA72-4**	Negative	56	59	0.007	0.931
	Positive	6	6		
**Preoperative CEA**	Negative	49	41	4.192	**0.041**
	Positive	14	26		
**Preoperative CTCs**	Negative	37	29	3.100	*0.078*
	Positive	26	38		
**Postoperative CA125**	Negative	33	39	2.483	0.115
	Positive	8	20		
**Postoperative CA19-9**	Negative	59	58	1.810	0.178
	Positive	4	9		
**Postoperative CA72-4**	Negative	60	59	2.160	0.142
	Positive	3	8		
**Postoperative CEA**	Negative	57	61	0.013	0.911
	Positive	6	6		
**Tumor recurrence**	No recurrence	54	48	8.073	**0.004**
	Recurrence	3	15		

Abbreviations: CRC, colorectal cancer; CTCs, circulating tumor cells; CEA, carcinoembryonic antigen; CA125, carbohydrate antigen 125; CA19-9, carbohydrate antigen 19-9; CA72-4, carbohydrate antigen 72-4. Note, Bold, indicates the association was significant; Italic, indicates a trend of association (0.5≤P<0.1).

**Table 3:** Effects of clinical basal characteristics, preoperative and postoperative blood tumor biomarkers on tumor recurrence survival in stage II-III CRC analyzed by univariate and multivariate Cox proportional hazard regression.

Parameters	Univariate analysis							Multivariate analysis					
	B	Wald	P-value	HR	95.0% CI			B	Wald	P-value	HR	95.0% CI	
**Gender (Female vs. Male)**	-0.157	0.111	0.739	0.855	0.339	2.154							
**Age (>62 vs.≤62 yrs)**	0.236	0.237	0.626	1.266	0.490	3.270							
**Tumor location (Colon vs. Rectum)**	0.225	0.202	0.653	1.252	0.470	3.338							
**T stage (T3-4 vs. T1-2)**	0.532	0.503	0.478	1.702	0.391	7.404							
**N stage (N1-2 vs. N0)**	1.057	4.471	**0.034**	2.879	1.080	7.672							
**TNM stage (III vs. II)**	1.301	6.112	**0.013**	3.674	1.309	10.306		1.331	6.218	**0.013**	3.786	1.330	10.780
**Tumor differentiation** **(Well/Moderate vs. Poor)**	-0.624	0.972	0.324	0.536	0.155	1.852							
**Preoperative CA125 (Positive vs. Negative)**	1.060	4.982	**0.026**	2.886	1.138	7.318							
**Preoperative CA19-9 (Positive vs. Negative)**	1.171	4.947	**0.026**	3.226	1.149	9.056							
**Preoperative CA72-4 (Positive vs. Negative)**	0.511	0.651	0.420	1.667	0.482	5.765							
**Preoperative CEA (Positive vs. Negative)**	0.344	0.506	0.477	1.410	0.547	3.639							
**Preoperative CTCs (Positive vs. Negative)**	-0.048	0.006	0.939	0.953	0.276	3.292							
**Postoperative CA125 (Positive vs. Negative)**	1.027	4.436	**0.035**	2.793	1.074	7.264							
**Postoperative CA19-9 (Positive vs. Negative)**	1.152	4.105	**0.043**	3.165	1.038	9.649							
**Postoperative CA72-4 (Positive vs. Negative)**	1.721	11.823	**0.001**	5.589	2.096	14.904		1.736	11.316	**0.001**	5.675	2.064	15.604
**Postoperative CEA (Positive vs. Negative)**	1.340	5.507	**0.019**	3.821	1.247	11.705							
**Postoperative CTCs (Positive vs. Negative)**	1.490	8.869	**0.003**	4.435	1.664	11.820		1.008	4.178	**0.041**	2.739	1.042	7.200

Abbreviations: CRC, colorectal cancer; CTCs, circulating tumor cells; CEA, carcinoembryonic antigen; CA125, carbohydrate antigen 125; CA19-9, carbohydrate antigen 19-9; CA72-4, carbohydrate antigen 72-4; HR, hazard ratio; CI, confidence interval. Note, Bold, indicates the association was significant.

**Table 4:** Comparison of tumor recurrence survival in stage II-III CRC patients stratified by different risk scores in prognostic models combining TNM stage, postoperative CA72-4 and postoperative CTCs or combining postoperative CA72-4 and postoperative CTCs analyzed by univariate and multivariate Cox proportional hazard regression.

Parameters	Univariate analysis							Multivariate analysis					
	B	Wald	P-value	HR	95.0% CI			B	Wald	P-value	HR	95.0% CI	
**Prognostic score 1 (TNM+CA72-4+CTCs)**													
Binary classification													
*High risk vs. Low risk*	1.774	13.442	**<0.001**	5.894	2.283	15.213							
Multiple classification		10.765	**0.005**										
*Moderate risk vs. Low risk*	1.918	3.267	*0.071*	6.810	0.851	54.517							
*High risk vs. Low risk*	2.998	8.090	**0.004**	20.053	2.540	158.304							
**Prognostic score 2 (CA72-4+CTCs)**													
Binary classification													
*High risk vs. Low risk*	1.837	10.492	**0.001**	6.279	2.066	19.083		1.807	10.146	**0.001**	6.092	2.004	18.521
Multiple classification		13.458	**0.001**						13.035	**0.001**			
*Moderate risk vs. Low risk*	1.692	8.381	**0.004**	5.429	1.727	17.069		1.666	8.133	**0.004**	5.289	1.684	16.616
*High risk vs. Low risk*	2.669	12.116	**<0.001**	14.427	3.210	64.847		2.647	11.725	**0.001**	14.111	3.101	64.201

Abbreviations: CRC, colorectal cancer; CTCs, circulating tumor cells; CA72-4, carbohydrate antigen 72-4; HR, hazard ratio; CI, confidence interval. Note, Bold, indicates the association was significant; Italic, indicates a trend of association (0.5≤P<0.1).

**Table 5:** Tumor recurrence rate in stage II-III CRC patients stratified by different risk scores in prognostic models combining TNM stage, postoperative CA72-4 and postoperative CTCs or combining postoperative CA72-4 and postoperative CTCs.

Parameters	Subgroups	Tumor recurrence		χ^2^	P-value
		No recurrence	Recurrence		
Prognostic model 1 (TNM+CA72-4+CTCs)					
Binary classification	Low risk	85	7	16.894	<0.001
	High risk	17	11		
Multiple classification	Low risk	46	1	16.651	<0.001
	Moderate risk	42	8		
	High risk	14	9		
Prognostic model 2 (CA72-4+CTCs)					
Binary classification	Low risk	72	4	15.412	<0.001
	High risk	30	14		
Multiple classification	Low risk	72	4	18.926	<0.001
	Moderate risk	28	11		
	High risk	2	3		

Abbreviations: CRC, colorectal cancer; CTCs, circulating tumor cells; CA72-4, carbohydrate antigen 72-4. Note, Bold, indicates the association was significant.

**Table 6:** Accuracy of postoperative CTCs and the constructed prognostic models in predicting tumor recurrence in stage II-III CRC

Parameters	AUC	Sensitivity (%)	Specificity (%)
**Postoperative CTCs**	0.673	61.11	73.53
**Prognostic model 1 (TNM+CA72-4+CTCs)**	0.722	61.11	83.33
**Prognostic model 2 (CA72-4+CTCs)**	0.742	77.78	70.59

Abbreviations: CRC, colorectal cancer; CTCs, circulating tumor cells; CA72-4, carbohydrate antigen 72-4; AUC, area under the receiver operating curves.

**Table 7:** Accuracy of postoperative CTCs and the constructed prognostic models in predicting tumor recurrence in stage II-III rectal cancer or colon cancer

Parameters	AUC	Sensitivity (%)	Specificity (%)
**Rectal cancer**			
Postoperative CTCs	0.507	50.00	51.35
Prognostic score 1 (TNM+CA724+CTCs)	0.739	66.67	81.08
Prognostic score 2 (CA724+CTCs)	0.671	66.67	67.57
**Colon cancer**			
Postoperative CTCs	0.769	100.00	53.85
Prognostic score 1 (TNM+CA724+CTCs)	0.715	58.33	84.62
Prognostic score 2 (CA724+CTCs)	0.778	83.33	72.31

Abbreviations: CRC, colorectal cancer; CTCs, circulating tumor cells; CA72-4, carbohydrate antigen 72-4; AUC, area under the receiver operating curves.

## References

[1] Bray F, Ferlay J, Soerjomataram I, Siegel RL, Torre LA, Jemal A (2018). Global cancer statistics 2018: GLOBOCAN estimates of incidence and mortality worldwide for 36 cancers in 185 countries. CA Cancer J Clin.

[2] Edwards BK, Ward E, Kohler BA, Eheman C, Zauber AG, Anderson RN (2010). Annual report to the nation on the status of cancer, 1975-2006, featuring colorectal cancer trends and impact of interventions (risk factors, screening, and treatment) to reduce future rates. Cancer.

[3] Hayashi M, Inoue Y, Komeda K, Shimizu T, Asakuma M, Hirokawa F (2010). Clinicopathological analysis of recurrence patterns and prognostic factors for survival after hepatectomy for colorectal liver metastasis. BMC Surg.

[4] Fidler IJ (2003). The pathogenesis of cancer metastasis: the 'seed and soil' hypothesis revisited. Nat Rev Cancer.

[5] de Albuquerque A, Kaul S, Breier G, Krabisch P, Fersis N (2012). Multimarker Analysis of Circulating Tumor Cells in Peripheral Blood of Metastatic Breast Cancer Patients: A Step Forward in Personalized Medicine. Breast Care (Basel).

[6] Riethdorf S, Fritsche H, Muller V, Rau T, Schindlbeck C, Rack B (2007). Detection of circulating tumor cells in peripheral blood of patients with metastatic breast cancer: a validation study of the CellSearch system. Clin Cancer Res.

[7] de Bono JS, Scher HI, Montgomery RB, Parker C, Miller MC, Tissing H (2008). Circulating tumor cells predict survival benefit from treatment in metastatic castration-resistant prostate cancer. Clin Cancer Res.

[8] Cohen SJ, Punt CJ, Iannotti N, Saidman BH, Sabbath KD, Gabrail NY (2008). Relationship of circulating tumor cells to tumor response, progression-free survival, and overall survival in patients with metastatic colorectal cancer. J Clin Oncol.

[9] Lu YJ, Wang P, Peng J, Wang X, Zhu YW, Shen N (2017). Meta-analysis Reveals the Prognostic Value of Circulating Tumour Cells Detected in the Peripheral Blood in Patients with Non-Metastatic Colorectal Cancer. Sci Rep.

[10] Jia S, Zhang R, Li Z, Li J (2017). Clinical and biological significance of circulating tumor cells, circulating tumor DNA, and exosomes as biomarkers in colorectal cancer. Oncotarget.

[11] Wang JY, Wu CH, Lu CY, Hsieh JS, Wu DC, Huang SY (2006). Molecular detection of circulating tumor cells in the peripheral blood of patients with colorectal cancer using RT-PCR: significance of the prediction of postoperative metastasis. World J Surg.

[12] Steinert G, Scholch S, Koch M, Weitz J (2012). Biology and significance of circulating and disseminated tumour cells in colorectal cancer. Langenbecks Arch Surg.

[13] Yang Y, Li J, Jin L, Wang D, Zhang J, Wang J (2017). Independent Correlation Between Ki67 Index and Circulating Tumor Cells in the Diagnosis of Colorectal Cancer. Anticancer Res.

[14] Allen-Mersh TG, McCullough TK, Patel H, Wharton RQ, Glover C, Jonas SK (2007). Role of circulating tumour cells in predicting recurrence after excision of primary colorectal carcinoma. Br J Surg.

[15] Bessa X, Pinol V, Castellvi-Bel S, Piazuelo E, Lacy AM, Elizalde JI (2003). Prognostic value of postoperative detection of blood circulating tumor cells in patients with colorectal cancer operated on for cure. Ann Surg.

[16] Koch M, Kienle P, Kastrati D, Antolovic D, Schmidt J, Herfarth C (2006). Prognostic impact of hematogenous tumor cell dissemination in patients with stage II colorectal cancer. Int J Cancer.

[17] Kust D, Samija I, Kirac I, Radic J, Kovacevic D, Kusic Z (2016). Cytokeratin 20 positive cells in blood of colorectal cancer patients as an unfavorable prognostic marker. Acta Clin Belg.

[18] Lu CY, Uen YH, Tsai HL, Chuang SC, Hou MF, Wu DC (2011). Molecular detection of persistent postoperative circulating tumour cells in stages II and III colon cancer patients via multiple blood sampling: prognostic significance of detection for early relapse. Br J Cancer.

[19] Sadahiro S, Suzuki T, Maeda Y, Yurimoto S, Yasuda S, Makuuchi H (2007). Detection of carcinoembryonic antigen messenger RNA-expressing cells in peripheral blood 7 days after curative surgery is a novel prognostic factor in colorectal cancer. Ann Surg Oncol.

[20] Sotelo MJ, Sastre J, Maestro ML, Veganzones S, Vieitez JM, Alonso V (2015). Role of circulating tumor cells as prognostic marker in resected stage III colorectal cancer. Ann Oncol.

[21] van Dalum G, Stam GJ, Scholten LF, Mastboom WJ, Vermes I, Tibbe AG (2015). Importance of circulating tumor cells in newly diagnosed colorectal cancer. Int J Oncol.

[22] Galizia G, Gemei M, Orditura M, Romano C, Zamboli A, Castellano P (2013). Postoperative detection of circulating tumor cells predicts tumor recurrence in colorectal cancer patients. J Gastrointest Surg.

[23] Rahbari NN, Aigner M, Thorlund K, Mollberg N, Motschall E, Jensen K (2010). Meta-analysis shows that detection of circulating tumor cells indicates poor prognosis in patients with colorectal cancer. Gastroenterology.

[24] Chou WC, Wu MH, Chang PH, Hsu HC, Chang GJ, Huang WK (2018). A Prognostic Model Based on Circulating Tumour Cells is Useful for Identifying the Poorest Survival Outcome in Patients with Metastatic Colorectal Cancer. Int J Biol Sci.

