# Supplementary material for: Prognostic models based on postoperative circulating tumor cells can predict poor tumor recurrence-free survival in patients with stage II-III colorectal cancer
Source: J Cancer. 2019 Jul 25;10(19):4552–63. doi: 10.7150/jca.30512 (PMC6746136; doi:10.7150/jca.30512)
Supplement: Supplementary file 1 — Supplementary tables. [file jcav10p4552s1.pdf]

Table S1. Baseline clinical characteristics of the enrolled patients with stage II-III CRC

**Parameters**	**Values**
Age (yrs.)	63.00 (56.00-72.25)
Gender (Male/Female)	76/54
Tumor location (Rectum/Colon)	47/83
T stage (T1-2/T3-4)	20/110
N stage (N0/N1-2)	77/53
TNM stage (II/III)	75/55
Tumor differentiation (Poor/Moderate or well)	12/116
Preoperative CA125 (U/ml)	3.325 (1.738-9.245)
Preoperative CA19-9 (U/ml)	9.500 (7.000-15.850)
Preoperative CA72-4 (U/ml)	9.355 (4.273-21.415)
Preoperative CEA (ng/ml)	2.200 (1.240-4.420)
Preoperative CTCs (number)	2.000 (1.000-4.000)
Postoperative CA125 (U/ml)	8.930 (6.340-15.280)
Postoperative CA19-9 (U/ml)	6.820 (2.698-15.118)
Postoperative CA72-4 (U/ml)	1.540 (0.958-3.433)
Postoperative CEA (ng/ml)	2.250 (1.383-3.363)
Postoperative CTCs (number)	4.000 (1.000-7.000)

Abbreviations: CRC, colorectal cancer; CTCs, circulating tumor cells; CEA, carcinoembryonic antigen; CA125, carbohydrate antigen 125; CA19-9, carbohydrate antigen 19-9; CA72-4, carbohydrate antigen 72-4.

Table S2. Associations of preoperative CA72-4 with basal characteristics, pre- and postoperative tumor biomarkers, and tumor recurrence in stage II-III CRC

**Parameters**	**Subgroups**	**Preoperative CA72-4**		**χ2**	**P-value**
		**Negative**	**Positive**		
**Gender**	Male	68	6	0.373	0.542
	Female	47	6		
**Age**	≤62 yrs	52	8	NA	0.225
	>62 yrs	63	4		
**Tumor location**	Rectum	41	6	0.960	0.327
	Colon	74	6		
**T stage**	T1-2	16	4	NA	*0.096*
	T3-4	99	8		
**N stage**	N0	68	6	0.373	0.542
	N1-2	47	6		
**TNM stage**	II	67	6	0.303	0.582
	III	48	6		
**Tumor differentiation**	Poor	11	1	NA	1.000
	Moderate/well	104	11		
**Preoperative CA125**	Negative	89	9	NA	0.728
	Positive	25	3		
**Preoperative CA19-9**	Negative	102	10	NA	0.634
	Positive	13	2		
**Preoperative CEA**	Negative	82	7	0.872	0.350
	Positive	33	5		
**Preoperative CTCs**	Negative	58	8	NA	0.369

	Positive	57	4		
**Postoperative CA125**	Negative	64	8	NA	0.721
	Positive	26	2		
**Postoperative CA19-9**	Negative	104	10	NA	0.354
	Positive	11	2		
**Postoperative CA72-4**	Negative	108	8	NA	**0.011**
	Positive	7	4		
**Postoperative CEA**	Negative	106	9	NA	0.087
	Positive	9	3		
**Postoperative CTCs**	Negative	56	6	0.007	0.931
	Positive	59	6		
**Tumor recurrence**	No recurrence	90	9	NA	0.393
	Recurrence	15	3		

Abbreviations: CRC, colorectal cancer; CTCs, circulating tumor cells; CEA, carcinoembryonic antigen; CA125, carbohydrate antigen 125; CA19-9, carbohydrate antigen 19-9; CA72-4, carbohydrate antigen 72-4.

Note: NA, not applicable and indicated the association was determined by Fisher's exact test. Bold, indicates the association was significant; Italic, indicates a trend of association (0.5≦P＜0.1).

Table S3. Associations of postoperative CA72-4 with basal characteristics, pre- and postoperative tumor biomarkers, and tumor recurrence in stage II-III CRC

**Parameters**	**Subgroups**	**Postoperative CA72-4**		**χ2**	**P-value**
		**Negative**	**Positive**		
**Gender**	Male	70	6	0.076	0.783
	Female	49	5		
**Age**	≤62 yrs	55	6	0.280	0.596
	>62 yrs	64	5		
**Tumor location**	Rectum	42	5	0.450	0.502
	Colon	77	6		
**T stage**	T1-2	16	4	NA	*0.066*
	T3-4	103	7		
**N stage**	N0	72	5	0.944	0.331
	N1-2	47	6		
**TNM stage**	II	69	6	0.049	0.825
	III	50	5		
**Tumor differentiation**	Poor	11	1	NA	1.000
	Moderate/well	106	10		
**Preoperative CA125**	Negative	93	7	1.330	0.249
	Positive	25	4		
**Preoperative CA19-9**	Negative	106	9	NA	0.615
	Positive	13	2		
**Preoperative CA72-4**	Negative	108	7	NA	**0.011**
	Positive	8	4		

**Preoperative CEA**	Negative	82	8	NA	0.546
	Positive	37	3		
**Preoperative CTCs**	Negative	58	8	NA	0.207
	Positive	61	3		
**Postoperative CA125**	Negative	67	5	4.320	**0.038**
	Positive	22	6		
**Postoperative CA19-9**	Negative	108	9	0.894	0.344
	Positive	11	2		
**Postoperative CEA**	Negative	110	8	4.669	**0.031**
	Positive	9	3		
**Postoperative CTCs**	Negative	60	3	2.160	0.142
	Positive	59	8		
**Tumor recurrence**	No recurrence	97	5	14.854	**<0.001**
	Recurrence	12	6		

Abbreviations: CRC, colorectal cancer; CTCs, circulating tumor cells; CEA, carcinoembryonic antigen; CA125, carbohydrate antigen 125; CA19-9, carbohydrate antigen 19-9; CA72-4, carbohydrate antigen 72-4.

Note: NA, not applicable and indicated the association was determined by Fisher's exact test. Bold, indicates the association was significant; Italic, indicates a trend of association (0.5≦P＜0.1).
